# Retraction: Facial mask detection using depthwise separable convolutional neural network model during COVID-19 pandemic

**DOI:** 10.3389/fpubh.2025.1726542

**Published:** 2025-10-23

**Authors:** Frontiers Editorial Office

The journal retracts the 07 March 2022 article cited above.

Following publication, the publisher has found evidence of peer review manipulation. As the scientific integrity of the article cannot be guaranteed and adhering to the recommendations of the Committee on Publication Ethics (COPE), the publisher therefore retracts the article. Please note that we have not confirmed whether all or any of the authors were aware of or involved in any form of misconduct or manipulation of the publication process.

This retraction was approved by the Chief Executive Editor of Frontiers. The authors received a communication regarding the retraction and had a chance to respond. This communication is recorded by the publisher.

